# Sub-acute systemic erythropoietin administration reduces ischemic brain injury in an age-dependent manner

**DOI:** 10.18632/oncotarget.9652

**Published:** 2016-05-29

**Authors:** Peter Thériault, Audrey Le Béhot, Ayman ElAli, Serge Rivest

**Affiliations:** ^1^ Neuroscience Laboratory, CHU de Québec Research Center and Department of Molecular Medicine, Faculty of Medicine, Laval University, Québec City, QC, Canada; ^2^ Neuroscience Laboratory, CHU de Québec Research Center and Department of Psychiatry and Neuroscience, Faculty of Medicine, Laval University, Québec City, QC, Canada

**Keywords:** ischemic stroke, erythropoietin, aging, inflammation, monocyte chemotactic protein-1, Gerotarget

## Abstract

Stroke is associated with neuroinflammation, neuronal loss and blood-brain barrier (BBB) breakdown. Thus far, recombinant tissue-type plasminogen activator (rtPA), the only approved treatment for acute ischemic stroke, increases the risk of intracerebral hemorrhage and is poorly efficient in disaggregating platelet-rich thrombi. Therefore, the development of safer and more efficient therapies is highly awaited. Encouraging neuroprotective effects were reported in mouse models of ischemic stroke following administration of erythropoietin (EPO). However, previous preclinical studies did not investigate the effects of EPO in focal ischemic stroke induced by a platelet-rich thrombus and did not consider the implication of age. Here, we performed middle cerebral artery occlusion by inducing platelet-rich thrombus formation in chimeric 5- (i.e. young) and 20- (i.e. aged) months old C57BL/6 mice, in which hematopoietic stem cells carried the green fluorescent protein (GFP)-tag. Recombinant human EPO (rhEPO) was administered 24 hours post-occlusion and blood-circulating monocyte populations were studied by flow cytometry 3 hours post-rhEPO administration. Twenty-four hours following rhEPO treatment, neuronal loss and BBB integrity were assessed by quantification of Fluoro-Jade B (FJB)-positive cells and extravasated serum immunoglobulins G (IgG), respectively. Neuroinflammation was determined by quantifying infiltration of GFP-positive bone marrow-derived cells (BMDC) and recruitment of microglial cells into brain parenchyma, along with monocyte chemotactic protein-1 (MCP-1) brain protein levels. Here, rhEPO anti-inflammatory properties rescued ischemic injury by reducing neuronal loss and BBB breakdown in young animals, but not in aged littermates. Such age-dependent effects of rhEPO must therefore be taken into consideration in future studies aiming to develop new therapies for ischemic stroke.

## INTRODUCTION

Stroke is the second cause of mortality worldwide after heart diseases and occurs following the interruption of blood circulation in brain by cerebral vessel burst (i.e. hemorrhagic stroke, 13% of cases) or occlusion (i.e. ischemic stroke, 87% of cases) [1] following an embolus or a local thrombus formation [2]. Thrombosis depends on local platelet aggregation and activation of coagulation factors, leading to the formation of a fibrin-rich clot [3]. Under physiological conditions, a fibrinolytic process is triggered in order to prevent the progression of fibrin thrombi, which is regulated by the interaction between endogenous tissue-type plasminogen activator (tPA), its inhibitors and the plasminogen/plasmin system [4, 5]. Accordingly, the administration of recombinant tPA (rtPA, Actilyse^®^) has been intensively studied both in animal models and patients [6, 7]. As vessel recanalization induced by rtPA administration improves functional outcome and reduces neurological deficits, it remains the sole acute ischemic stroke treatment [8]. Nonetheless, rtPA presents some concerning limitations such as poor efficiency in disaggregating platelet-rich thrombi [9, 10], increased risk of intracerebral hemorrhage [8] and tight administration time-window (i.e. 4.5 hours) [11, 12]. Altogether, these factors restrain its application as less than 10% of ischemic stroke patients are thrombolysed [13]. Therefore, in order to restore vessel patency after ischemic stroke and improve functional outcomes, it is imperative to develop safer and more efficient therapeutic strategies.

Erythropoietin (EPO), a member of the hematopoietic cytokine superfamily, modulates hematopoiesis and stimulates erythropoiesis in the bone marrow (BM) [14, 15]. During metabolic stress, EPO is produced locally in different organs, such as the brain [16], and acts as a multifunctional protective molecule [17, 18]. Therefore, it was speculated that EPO administration might constitute a promising therapeutic approach in brain disorders, such as ischemic stroke [19]. Indeed, a number of findings demonstrated *in vivo* robust neuroprotective properties of exogenous EPO [20]. More precisely, systemic EPO administration has been shown to reach the ischemic brain, activating anti-apoptotic and anti-inflammatory signaling in neurons and glial cells [20], thus reducing cerebral damage [21, 22]. As such, this suggests acute and chronic actions for EPO in the ischemic brain.

Although encouraging results were reported, the effects of EPO seem to depend on the time and the animal models of stroke [23]. First, despite age is a major contributor in the prevalence, incidence and outcome of ischemic stroke [24], most *in vivo* studies were performed in young animals (i.e. 2- to -6-months old). Second, EPO administration is mainly performed before arterial obstruction [25] or at time of reperfusion [26], contrasting with thrombolysis conditions observed in patients. Finally, arterial occlusion in animal models is widely induced by an intraluminal filament or electrocoagulation, whereas in patients, occlusion is due to thrombus formation by embolism or local occlusive thrombosis [2].

Our study is based on the urge of developing new therapeutical approaches that consider age in ischemic stroke models that are more closely associated to the human pathophysiology. Here, we observed neuroprotective effects following sub-acute recombinant human EPO (rhEPO) administration in an ischemic stroke model based on platelet-rich thrombus formation [27], using chimeric 5- (i.e. young) and 20- (i.e. aged) months old mice.

## RESULTS

### rhEPO administration limits neuronal loss and BBB breakdown in young animals, but not in aged ones

**Figure 1:**
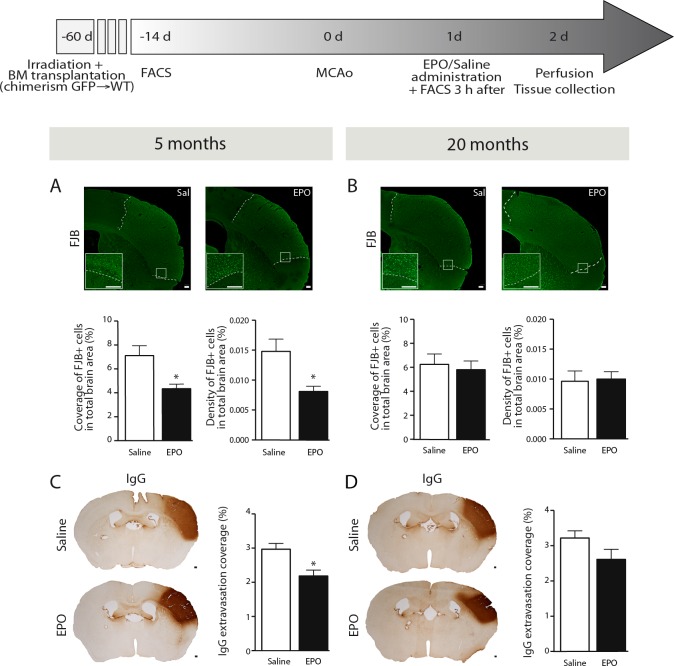
rhEPO administration limits neuronal loss and BBB breakdown in ischemic injury Timeline of experimentation (grey arrow). Representative images of FJB staining and respective stereological quantifications of FJB-positive cells coverage and density (relative % of the total brain area) of saline- or EPO-treated 5- (i.e. young) **A.** and 20- (i.e. aged) **B.** months old animals. Representative images of IgG staining and respective stereological quantification of IgG extravasation coverage area (relative % of the total brain area) of saline- or EPO-treated 5- **C.** and 20- **D.** months old animals. Values are expressed as means ± SEM. Statistical analyses were performed using an unpaired *t-test*. * = *p* < 0.05 significant difference compared to saline. n = 8-9 mice per group. Scale bars (A-D) = 150 μm. Abbreviations: BBB: blood-brain barrier; BM: bone marrow; d: day; EPO: erythropoietin; FACS: flow cytometry; FJB: Fluoro-Jade B; GFP: green fluorescent protein; h: hour; IgG: immunoglobulins G; MCAo: middle cerebral artery occlusion; Sal: saline; WT: wild type.

In order to evaluate the impact of sub-acute rhEPO administration on neuronal loss following ischemic injury, we quantified FJB-positive neuronal cells by stereological analysis in brains of 5- (i.e. young) and 20- (i.e. aged) months old mice. We observed a significant reduction of FJB-positive cells coverage (Figure 1A, left) and density (Figure 1A, right) in brains of rhEPO-treated young animals in comparison to saline-treated ones, while no changes were observed neither in FJB-positive cells coverage (Figure 1B, left) or density (Figure 1B, right) in aged littermates. Moreover, in order to assess BBB integrity, we measured serum IgG extravasation. We observed that rhEPO significantly reduces IgG extravasation in brains of young animals in comparison to saline-treated ones (Figure 1C). However, no changes were observed in aged littermates (Figure 1D). These results suggest that rhEPO limits neuronal loss and BBB breakdown in young animals, while no effects were observed in aged ones.

### rhEPO administration does not affect blood-circulating monocyte populations

**Figure 2:**
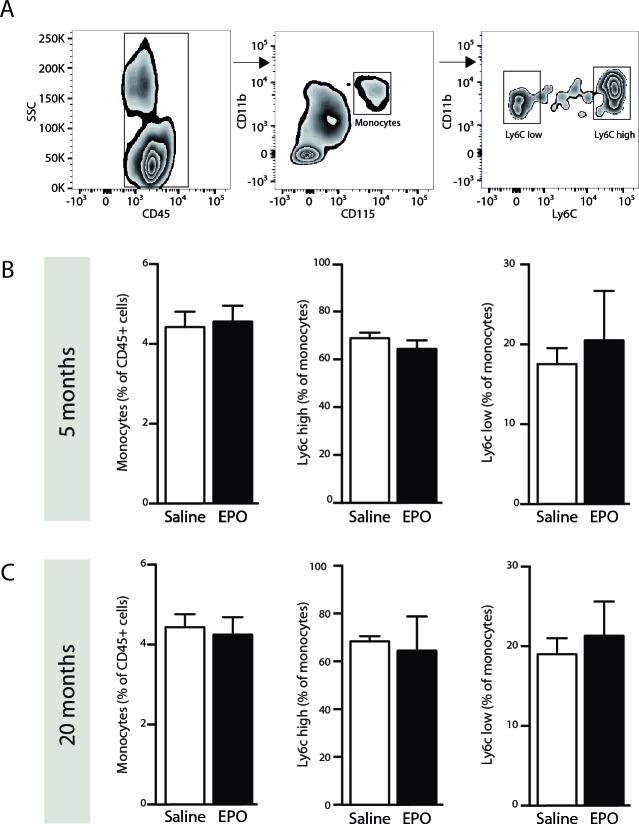
rhEPO administration does not affect blood-circulating monocyte populations Gating strategy **A.** to study populations of blood-circulating monocytes; leukocytes (CD45+, left panel), total monocytes (CD45+/CD11b+/CD115+, middle panel), pro-inflammatory (Ly6C^high^) and patrolling (Ly6C^low^) monocytes (right panel). **B.**, **C.** Frequencies of total monocytes (relative % of CD45-positive cells; left graph), Ly6C^high^ subset (relative % of total monocytes; middle graph) and Ly6C^low^ subset (relative % of total monocytes; right graph) in saline- or EPO-treated 5- (B) and 20- (C) months old animals. Values are expressed as means ± SEM. Statistical analyses were performed using an unpaired *t-test*. *n* = 8-9 mice per group. Abbreviations: EPO: erythropoietin; Sal: saline.

As mentioned, EPO is a hematopoietic cytokine that can modulate hematopoiesis [15], which can affect the production of bone marrow-derived cells (BMDC) such as monocytes. Therefore, 3 hours post-injection of rhEPO, we assessed by FACS analysis blood-circulating monocyte (CD45+CD11b+CD115+) frequencies, including pro-inflammatory (Ly6C^High^) and patrolling (Ly6C^Low^) subsets [28], in freshly isolated blood samples from young and aged mice (Figure 2A). Interestingly, we did not observed any changes in frequencies of neither total monocyte nor Ly6C^High^ and Ly6C^Low^ subsets following rhEPO administration, when compared to saline-treated ones, in both young and aged mice (Figure 2B, 2C respectively). These results indicate that systemic rhEPO administration does not affect blood-circulating monocyte population frequencies, regardless of age.

### rhEPO administration limits BMDC infiltration in the brain parenchyma of young animals, while it remains unchanged in aged littermates

**Figure 3:**
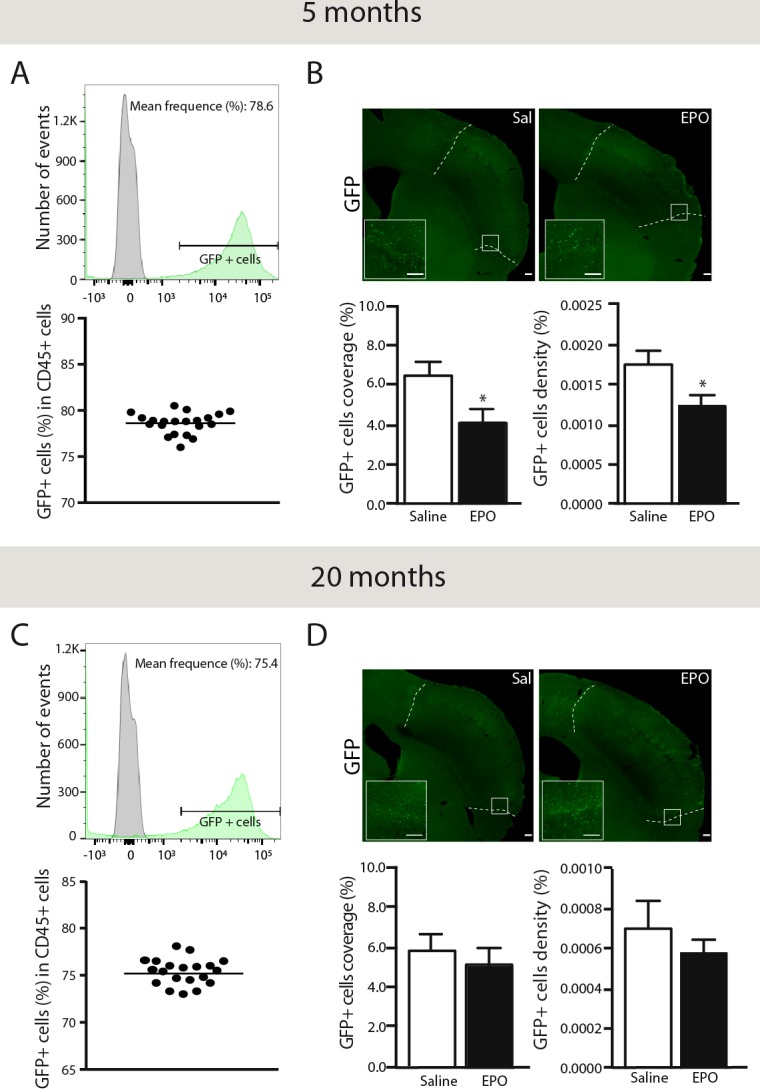
rhEPO limits brain infiltration of BMDC in the ischemic injury Representative histograms overlay (**A. C.** upper panel) showing the gating strategy for GFP-positive cells (green) relatively to GFP-negative cells derived from WT controls (grey). Representative graphs (A, C lower panel) showing frequencies of GFP-positive cells (relative % of CD45-positive cells) in 5- (A) and 20- (C) months old (GFP-WT) chimeric mice. Representative photomicrographs (**B.**, **D.** upper panels) and respective stereological quantifications of GFP-positive cells coverage and density (relative % of the total brain area) in saline- or EPO-treated 5- (B, lower panels) and 20- (D, lower panels) months old animals. Values are expressed as means ± SEM. Statistical analyses were performed using an unpaired *t test.* * = *p* < 0.05 significant difference compared to saline. n = 8-9 mice per group. Scale bars: B, D = 150 μm. Abbreviations: GFP: green fluorescent protein; EPO: erythropoietin; Sal: saline.

Chimeric mice (GFP→WT) were generated in order to assess rhEPO's anti-inflammatory effects on BMDC brain infiltration (Figure 1, timeline). Forty-six days post-transplantation, we determined chimerism by FACS in freshly isolated blood samples from young (Figure 3A) and aged chimeric mice (Figure 3C). Thereafter, we assessed brain infiltration of GFP+ BMDC *via* quantification of endogenous monocyte-like GFP+ cells in brains of young (Figure 3B) and aged mice (Figure 3D). We observed a significant reduction of GFP-positive cells coverage area (Figure 3B, left) and density (Figure 3B, right) in brains of rhEPO-treated young mice in comparison to saline-treated ones. However, when comparing the two groups, no changes were detected in aged mice (Figure 3D). Thus, rhEPO administration likely limits the entry of BMDC in the brain parenchyma of young animals, while no changes were observed in aged littermates.

### rhEPO administration reduces microglial cells recruitment and monocyte chemotactic protein-1 (MCP-1) protein levels in the ischemic brain of young animals, without any changes in aged ones

**Figure 4:**
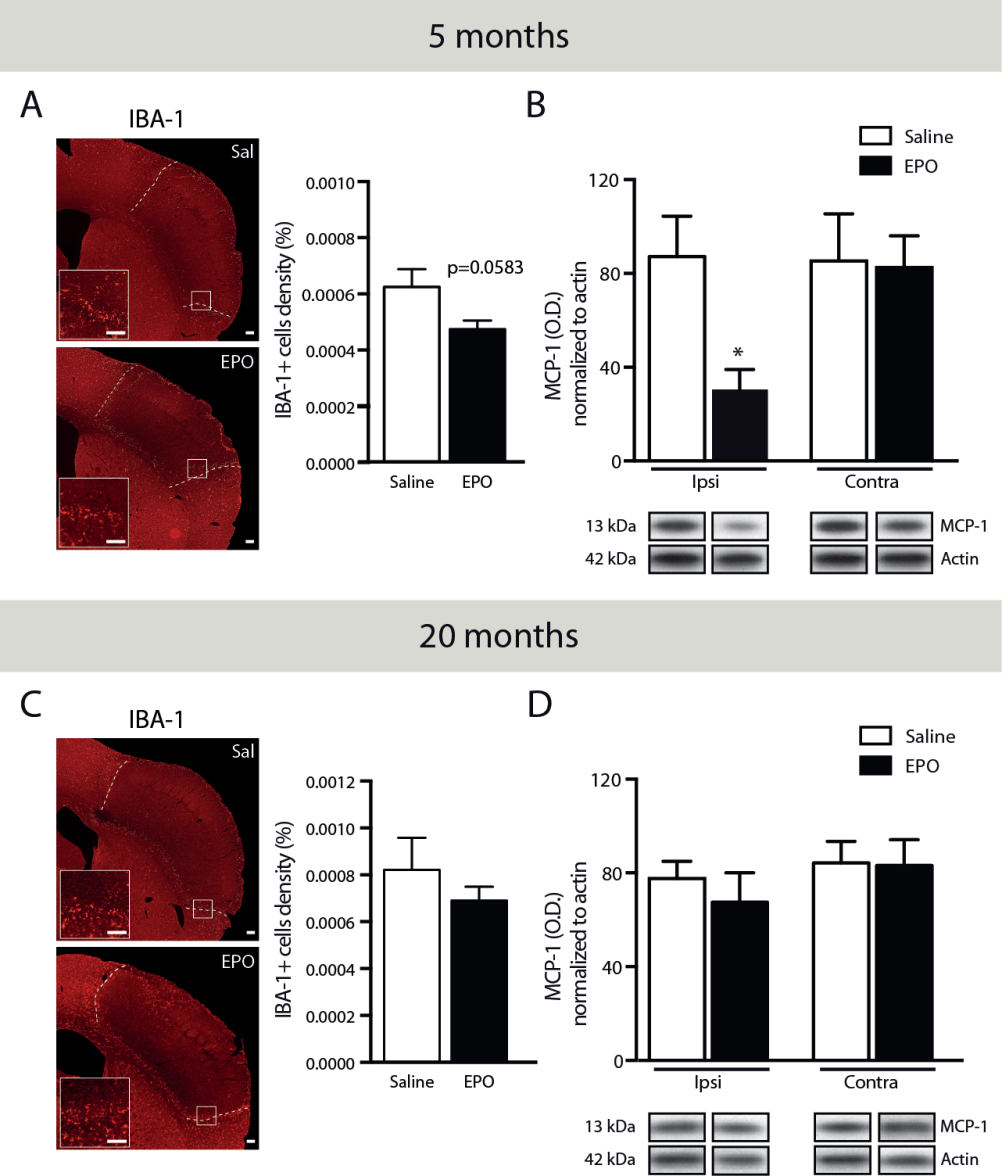
rhEPO administration limits microglial cell recruitment and reduces MCP-1 protein levels in ischemic injury Representative photomicrographs of IBA-1 immunostaining and respective stereological quantification of microglial cells density (relative % of the total brain area) of saline- or EPO-treated 5- **A.** and 20- **C.** months old animals. Quantifications by western blot analyses of brain MCP-1 protein levels normalized to β-actin protein levels in ischemic (ipsilateral hemisphere) in comparison to healthy tissue (contralateral hemisphere) of saline- or EPO-treated 5- **B.** and 20- **D.** months old animals. Represented bands are cropped blots derived from the same experiment. Values are expressed as mean ± SEM. Statistical analyses were performed using an unpaired *t*-test. * = *p* < 0.05 significant difference compared to saline. *n* = 8-9 mice per group in A and C, *n* = 4 mice per group in B and D. Scale bars: A, C = 150 μm. Abbreviations: Contra: contralateral, EPO: erythropoietin; IBA-1: ionized calcium-binding adapter molecule 1; Ipsi: ipsilateral, MCP-1: monocyte chemotactic protein-1; OD: optical density; Sal: saline; WT: wild-type.

Next, we evaluated the effects of rhEPO systemic administration on microglial cells response following ischemic injury. As such, we quantified IBA-1-positive cells by stereological analysis in brains of young (Figure 4A) and aged mice (Figure 4C). Interestingly, we observed a reduction, although statistically not significant (*p* = 0.0583), of IBA-1-positive cells density in brains of rhEPO-treated young mice in comparison to saline-treated littermates (Figure 4A, right). However, no changes were found either in rhEPO- or saline-treated aged mice (Figure 4C, right). Additionally, in order to elucidate the mechanism involved in the reduction of BMDC brain infiltration following rhEPO treatment, we investigated MCP-1 (also named chemokine (C-C motif) ligand 2 or CCL2) production, which is a chemokine involved in BMDC (e.g. monocytes) mobilization and recruitment [29]. Therefore, we measured MCP-1 protein levels by western blot analyses in brains of young (Figure 4B) and aged mice (Figure 4D). We detected a significant reduction of MCP-1 protein levels in the ipsilateral hemisphere (i.e. ischemic injury) of rhEPO-treated young mice in comparison to saline-treated ones, while no changes were observed in the contralateral hemisphere (i.e. healthy tissue) of neither rhEPO-treated nor saline-treated mice (Figure 4B). Moreover, no changes were observed in the ipsilateral or contralateral hemispheres in aged mice (Figure 4D). Altogether, these results suggest that rhEPO administration limits microglial cell activation and recruitment in ischemic injury, which consequently reduces MCP-1 production, thus preventing BMDC infiltration in the ischemic brain of young animals, which is not observed in aged animals.

## DISCUSSION

In this study, we evaluated the impact of sub-acute systemic rhEPO administration following ischemic stroke induced by a platelet-rich thrombus, in 5- (i.e. young) and 20- (i.e. aged) months old animals. Our results indicate that rhEPO administration reduces ischemic injury through its anti-inflammatory properties, limiting neuronal loss and BBB breakdown in an age-dependent manner. More precisely, in young animals, rhEPO significantly attenuates pro-inflammatory response by limiting brain infiltration of BMDC, which is accompanied by a reduced recruitment of microglial cells and decreased MCP-1 productionin the ischemic brain. However, these beneficial effects were not observed in aged animals, suggesting that age-related factors could abolish responsiveness to rhEPO.

In this regard, the majority of focal ischemic stroke models are based on young animals, although age profoundly affects several parameters, including arterial remodeling [30], BBB integrity, glial cells activation, neurogenesis and apoptosis [31, 32], which increase the susceptibility to brain injury [4, 31]. Therefore, large artery intracranial occlusive disease is considered as the most common subtype of ischemic stroke worldwide [2]. In this pathology, intracranial atherosclerotic lesions are responsible for both artery-to-artery embolisms and *in situ* occlusive thrombosis, leading to *in situ* formation of platelet-rich thrombi [2]. These findings underline an important role of age in stroke pathogenesis and thereby, the development of therapeutic strategies.

As such, besides its lack of efficiency for treatment of patients presenting platelet-rich thrombi [9], rtPA treatment increases the risk of intracerebral hemorrhage and presents potential neurotoxic effects, which greatly limit its clinical usage [7]. Therefore, we investigated a novel therapeutic approach based on EPO's cytoprotective effects [20], using an ischemic stroke model induced by platelet-rich thrombus [27]. As mentioned, we reported reduced neuronal loss and BBB breakdown in young rhEPO-treated animals in comparison to saline-treated littermates, 24 hours post-occlusion. In previous *in vivo* studies with ischemic mice, EPO was administered at earlier time points. For instance, Wang et al. showed that when administered 2 hours after arterial occlusion (i.e. at beginning of reperfusion), EPO reduces the infarct volume, oedema, BBB leakage and neurologic deficits as soon as 24 hours post-administration [26]. When injected before ischemic stroke [25] or during reperfusion [33], effectiveness of EPO reveals a larger therapeutic time-window when compared to rtPA, thus suggesting that rhEPO administration should be an effective treatment with a large time window following ischemic stroke, at least in young patients. As such, when administered in patients within 8 hours following ischemic stroke and re-administered 24 and 48 hours later, rhEPO is associated with an improvement in the clinical outcome one month after-treatment [34]. In addition, another clinical study reported a significant improvement of the long-term (i.e. 5 years) neurological outcomes of EPO-treated patients, using two consecutive doses of EPO (i.e. 5,000 IU/dose) administered at 48 hours and 72 hours following ischemic stroke [35], thus suggesting that EPO is an effective treatment for acute ischemic stroke. However, it has been reported that EPO exacerbates cerebral oedema, BBB leakage, and extracellular matrix breakdown in thrombolysed mice [36], suggesting that ischemic stroke patients may be potential candidates for rhEPO treatment, when not suitable for thrombolysis [35].

Our study revealed a neuroprotective role for EPO, through its anti-inflammatory properties, in the context of ischemic stroke induced by a platelet-rich thrombus. Although beneficial, animals’ responsiveness to EPO treatment is likely age-dependent since its neuroprotective effects are abolished with age. Thus, it is crucial to consider ageing and comorbidities in order to adapt ischemic stroke treatment for each patient. Therefore, further preclinical studies aiming at developing new therapeutic strategies for ischemic stroke must consider age in animal models, in order to better translate experimental findings into clinics.

## MATERIALS AND METHODS

Complete details of materials and methods are described in online supplement as supplemental methods.

### Animals

Experiments were performed according to the Canadian Council on Animal Care guidelines, as administered by the Laval University Animal Welfare Committee. Three- (i.e. young) and 18- (i.e. aged) months old adult male wild-type (WT; C57BL/6J) and age-matched green fluorescent protein [GFP^+/−^; C57BL/6-Tg (CAG-EGFP) Osb/J)] mice were used.

### Chimeric mouse conditioning and BM transplantation

WT chimeric mice were generated by transplanting BM cells isolated from GFP^+/−^ mice in irradiated WT mice (GFP→WT), as previously described [37]. The chimerism level was assessed by flow cytometry (FACS) analysis 6 weeks after BM transplantation. Experimental protocol was initiated 2 weeks later.

### Experimental design

Five- (i.e. young) and 20- (i.e. aged) months old C57/Bl6 chimeric (GFP→WT) mice were used (see timeline in Figure 1). Mice at 3 and 18 months of age were exposed to whole body irradiation prior to GFP-tagged BM-derived cells (BMDC) transplantation (−60 d) and chimerism was assessed (−14 d) by FACS analyses. Then, thrombosis-induced occlusion of the middle cerebral artery (MCAo) was induced by focal application of 30% ferric chloride (FeCl_3_) solution, thus inducing ischemic injury (0 d). rhEPO administration was performed 24 hours following ischemic stroke and blood-circulating monocyte populations were assessed 3 hours post-injection (1 d). Finally, animals were sacrificed 24 hours post-treatment for immunostaining and biochemical analyses (2 d).

### Focal cerebral ischemia and rhEPO administration

Thrombus formation was induced by 30% FeCl_3_ (Sigma-Aldrich) application on the middle cerebral artery (MCA) [38]. rhEPO (2500 IU/kg, EPREX^®^ 4000, Janssen) or vehicle (i.e. saline solution) was intraperitoneally injected 24 hours post-ischemic stroke.

### FACS analysis

To study monocyte populations in the blood circulation, facial vein blood was collected 3 hours post-rhEPO or saline administration. FACS analyses were performed as previously described [39].

### Tissue collection

Animals were euthanized 24 hours post-rhEPO or saline administration (i.e. 48 hours post-ischemic stroke). Brains were removed, cut into 25 μm-thick coronal sections using a freezing microtome (Leica Microsystems), collected in anti-freeze solution prior to histological analyses.

### Fluoro-Jade B staining and immunofluorescence

Neuronal death was assessed on coronal sections by Fluoro-Jade B (FJB) staining as described previously [40]. Microglial cells were detected by ionized calcium-binding adapter molecule-1 (IBA-1) immunofluorescent staining, as described previously [41]. Sections were mounted onto Micro Slides Superfrost® Plus and coverslipped with fluoromount G. Quantification of FJB^+^, IBA-1^+^, GFP^+^ cellular density and coverage area were performed by unbiased stereological analyses [42].

### Histochemical immunostaining

Blood-brain barrier (BBB) breakdown was assessed by immunoglobulins G (IgG) immunostaining ^31^ and sections were digitized prior to quantification of IgG^+^ coverage area (i.e. extravasation) using ImageJ software.

### Western blot analysis

Protein lysates were obtained from dissection of ischemic (ipsilateral hemisphere) and healthy (contralateral hemisphere) tissues and subjected to SDS-PAGE prior to western blot analysis. Anti-mouse monocyte chemotactic protein-1 (MCP-1) (1:1000; Cell Signaling) and anti-mouse β-actin (1:40000; EMD Millipore) primary antibodies were used prior to detection by horseradish peroxidase (HRP)-conjugated secondary antibodies and revelation by enhanced chemiluminescence plus (ECL) solution (GE Healthcare Life Sciences). Blots were digitized and proteins level were densitometrically analyzed with ImageJ software.

### Statistical analysis and image preparation

Results are expressed as mean ± SEM. Statistical analyses were performed using standard two-tailed unpaired *t*-test for comparison between 2 groups (saline- or rhEPO-treated groups), using GraphPad Prism software. All panels presented were assembles using Adobe Photoshop CS5 (version 12.0.4) and Adobe Illustrator CS5 (version 15.0.2).

## SUPPLEMENTARY MATERIAL AND REFERENCES


