# Supplementary material for: Sub-acute systemic erythropoietin administration reduces ischemic brain injury in an age-dependent manner
Source: Oncotarget. 2016 May 29;7(24):35552–61. doi: 10.18632/oncotarget.9652 (PMC5094944; doi:10.18632/oncotarget.9652)
Supplement: Supplementary file 1 [file oncotarget-07-35552-s001.pdf]

Sub-acute systemic erythropoietin administration reduces ischemic brain injury in an age-dependent manner

Supplementary Material

Methods

Animals

Experiments were conducted according to the Canadian Council on Animal Care guidelines, as managed by the Laval University Animal Welfare Committee. All efforts were made to reduce the number of animals used and to avoid suffering. Three (i.e. young) and 18- (i.e. aged) months old adult male C57BL/6J mice (wild type, WT) and age-matched mice expressing the green fluorescent protein [$GFP^{+/-}$, C57BL/6-Tg (CAG-EGFP)Osb/J)] were used to generate chimeras. Mice were housed 3 to 5 per cage and acclimated to standard laboratory conditions (12 hours light/dark cycle; lights on at 7:15 A.M. and off at 7:15 P.M.) with *ad libitum* access to chow and water. Following randomization, all experimental procedures and analyses were performed blind to animal group and treatment status. No mortality was induced.

Chimeric mouse conditioning and bone marrow transplantation

WT chimeric mice were generated by transplanting bone marrow-derived cells (BMDC) isolated from $GFP^{+/-}$ mice into irradiated C57BL/6 WT mice, as previously described [1]. A week before irradiation, 3- and 18- months old WT mice were given irradiated food and water containing a commercially available mix of antibiotics (SEPTRA, GlaxoSmithKline). They were then exposed to a 10 gray total-body irradiation using a cobalt-60 source (Theratron-780 model, MDS Nordion) and transferred for 1 week to sterile cages with irradiated food and fresh antibiotic-supplemented water. Twenty-four hours after irradiation, age-matched $GFP^{+/-}$ donor mice were killed by cervical dislocation under deep anesthesia (5 minutes, 2.5% isoflurane, AbbVie in 2.5% O_2). Their femurs and tibias were aseptically dissected and their bone marrow (BM) was flushed with Dulbecco's phosphate-buffered saline (DPBS, Sigma-Aldrich) with Ca^{2+}/Mg^{2+}, complemented with 5% fetal bovine serum (FBS, Sigma-Aldrich). BMDCs were filtered through a 40 µm nylon mesh (Falcon®, Corning Inc, Life Sciences), washed 3 times in DPBS with Ca^{2+}/Mg^{2+} (centrifuging at 300g for 10 minutes between washes) and counted with a hematocytometer. BDMCs (1.5×10^7) diluted in 200µl of in DPBS with Ca^{2+}/Mg^{2+} were injected into the tail vein of WT recipient mice. To allow injected-BMDC to repopulate the hematopoietic system, the chimerism level was assessed by flow cytometry (FACS) analysis 8 weeks after transplantation and 2 weeks later, when mice were 5 (i.e. young) and 20 (i.e. aged) months of age (n = 8-10 per group), experimental protocol was initiated.

Focal Cerebral Ischemia and recombinant human EPO (rhEPO) administration

Chimeric mice were deeply anesthetized (5 minutes, 2.5% isoflurane in 2.5% O_2) and placed on a heating pad in a stereotaxic device (Kopf Instruments) equipped with an isoflurane mask to maintain anesthesia (2% isoflurane in 1.5% O_2) during surgery. Through a skin incision between the right eye and ear, the masseter muscle was excised and a small craniotomy (diameter, 1 mm) was performed on the parietal bone in order to expose the right middle cerebral artery (MCA). A piece of Whatman™ filter paper strip soaked in freshly prepared ferric chloride ($FeCl_3$, 30%; Sigma-Aldrich) was applied on the intact dura mater [2] for 10 minutes, leading to clot formation and subsequent MCA occlusion (MCAo). To stabilize the thrombus, anesthesia was maintained for 1 hour. Then, animals were allowed to recover on the heating pad until they woke up and they returned to their home cage. Signs of pain or distress were regularly assessed and no animal was euthanized because of suffering. rhEPO (2500

IU/kg, EPREX® 4000, Janssen) solution was diluted in saline (NaCl, 0.9%) and administered by intraperitoneal (i.p.) injection 24 hours post-MCAo. Control animals received saline (NaCl, 0.9%).

FACS analysis

To assess blood-circulating monocyte populations, facial vein blood was collected 3 hours following administration of rhEPO or saline in EDTA coated vials (Microvette® K3E, Sarstedt). Flow cytometry analysis was performed as previously described [3]. Briefly, 50 μL of total blood were incubated on ice in the dark for 15 minutes with 4 μL of the anti-CD16/CD32 antibody (mouse Fc block, BD Biosciences) diluted in 11 μL of DPBS without Ca^{2+}/Mg^{2+}. Keeping the samples on ice and in the dark, the cells were stained with V500-conjugated anti-CD45 antibody (1:100; BD Biosciences), Alexa Fluor® 700-conjugated anti-CD11b antibody (1:100; eBioscience, San Diego, CA, USA), allophycocyanin (APC)-conjugated anti-CD115 antibody (1:100, eBioscience), phycoerythrin (PE)-conjugated anti-Ly6G antibody (1:100; BD Biosciences), V450-conjugated anti-Ly6C antibody (1:100; BD Biosciences) for 45 minutes. Red blood cells were lysed with 1.5 mL of 1X BD Pharm Lyse™ (BD Biosciences), accordingly to manufacturer's protocol. After hemolysis, remaining cells were washed with DPBS without Ca^{2+}/Mg^{2+} by centrifugation (350g, 8 minutes, 4°C) and resuspended in equal volumes of DPBS without Ca^{2+}/Mg^{2+}. Finally, cells were analyzed using a LSR II flow cytometer (BD™, BD Biosciences) and data was acquired with BD FACS Diva software (version 6.1.2, BD Biosciences). The results were compiled using FlowJo software (Version 7.6.1, Tree Star Inc).

Tissue collection

Twenty-four hours following treatment (rhEPO or saline), chimeric mice were deeply anesthetized via an i.p. injection of a mixture of ketamine hydrochloride and xylazine (100/10 mg/kg, Vetalar, Bioniche). For biochemical analyses, mice were transcardially perfused with ice-cold saline solution (0.9%, Sigma-Aldrich) by using a peristaltic pump. Brains were immediately removed and frozen in dry ice prior to storage at -80°C. For immunofluorescence and histochemical analysis, transcardiac perfusion was performed with ice-cold saline solution (0.9%, Sigma-Aldrich) followed by 4% paraformaldehyde (PFA, Electron Microscopy Sciences) solution (in 0.1 M PBS, pH 7.4) by using a peristaltic pump. Brains were removed and immersed overnight at 4°C in a 4% PFA solution containing 20% sucrose. Fixed brains were frozen with dry ice/ethanol mixture, mounted on a freezing-microtome (Leica SM 2000R, Leica Microsystems) and cut into 25 μm-thick coronal sections. Every section was collected starting from the end of the olfactory bulb to the end of the cerebral cortex, placed in a tissue cryoprotectant solution containing 0.05 M PBS (pH 7.3), 30% ethylene glycol, 20% glycerol and stored at -20°C until analysis.

Immunofluorescence

Free-floating sections were washed in phosphate-buffered saline (KPBS, 50 mM, Sigma-Aldrich, 3 x 10 minutes) and incubated for 30 minutes at room temperature (RT) in blocking solution containing 0.4% Triton X-100 (Sigma-Aldrich), 1% bovine serum albumin (BSA, Sigma-Aldrich) and 4% goat serum (Cederlane). To reveal microglia, sections were incubated overnight at 4°C with a primary antibody against the anti-ionized calcium binding adapter molecule-1 (IBA-1; 1:2000; Wako Chemicals) in 0.5 X blocking solution. The following day, after washes in KPBS, sections were incubated 2 hours with the Cy™3-conjugated anti-immunoglobulin G (IgG) secondary antibody (1:1000; Jackson Immunoresearch Laboratories) at RT, covered from light. Sections were then washed in KPBS, mounted onto Micro Slides Superfrost® Plus (VWR International) and coverslipped with Fluoromount-G (Electron Microscopy Sciences) [4].

Fluoro-Jade B staining

Free-floating brain sections were washed in potassium KPBS (3 x 10 minutes) at RT, mounted on Micro Slides Superfrost® Plus, air-dried and fixed with 4% PFA, pH 7.4 for 20 minutes. To reveal degenerating neurons, following washes in KPBS (2 x 5 minutes) and steps of dehydratation/rehydratation, the slides were immersed in 0.006% potassium permanganate (MP Biomedicals) for 5 minutes. After further rinsing, they were stained in 0.0004% Fluoro-Jade B solution (FJB, EMD Millipore) for 10 minutes. Sections were finally rinsed, air-dried overnight in the dark, cleared in xylene (3 x 2 minutes) and coverslipped with distyrene plasticizer xylene mounting medium (DPX, Electron Microscopy Sciences) [5].

Histochemical immunostaining

To reveal blood-brain barrier leakage, free-floating sections were washed in KPBS (3 × 10 minutes), incubated overnight at RT with a biotin-conjugated anti- immunoglobulines G (IgG) secondary antibody (1:1500, Vector Laboratories) then detected using the avidin peroxydase kit (Vectastain ABC kit, Vector Laboratories) and 3.3'-diaminobenzidine (DAB, 0.5 mg/ml, Sigma-Aldrich), following the manufacturer's instructions. Sections were mounted onto Micro Slides Superfrost® Plus, dehydrated and coverslipped with DPX [4].

Protein extraction

WT ischemic (i.e. ispsilateral hemisphere) and healthy (i.e. contralateral hemisphere) brain tissues were homogenized and lysed in NP-40 lysis buffer supplemented with 5% protease inhibitor cocktail and 1% phosphatase inhibitor cocktail 2 (Sigma-Aldrich). Lysate samples were sonicated over 2 cycles lasting 20 seconds each at 4°C at 40% power (Sonic dismembrator, model 100, Fisher Scientific). Protein concentration was determined using the Quantipro BCA assay kit (Sigma-Aldrich) according to the manufacturer's protocol. Absorbance was acquired with a microtiter plate reader (SpectraMax 340PC, Molecular Devices) and analysed using SOFTmax Pro3.1.1 software (Molecular Devices).

Western blot analysis

10 µg of proteins were diluted in 2X SDS loading buffer and heated (95°C, 5 minutes). Samples were migrated on a 4–15% precast SDS-polyacrylamide gel (Bio-Rad). Separated proteins were then transferred onto a PVDF membrane followed by western blot analysis. After incubation in blocking solution constituted of 5% skim milk diluted in 0.1 M tris-buffered saline and 0.05% Tween X-100 (TBS-T), membranes were incubated with the following primary antibodies: anti-monocyte chemotactic protein-1 (MCP-1, 1:1000; MCP-1, Cell Signaling Technology Inc) and anti-β-actin (1:40000; EMD Millipore). Primary antibodies were detected using horseradish peroxidase (HRP)–conjugated secondary antibodies (1:5000) in 5% skim milk diluted in TBS-T and revealed by enhanced chemiluminescence plus (ECL) solution (GE Healthcare Life Sciences). Blots were digitized, densitometrically analyzed with ImageJ image analysis software (NIH software, version 1.45s, National Institute of Health) and corrected for protein loading by means of β-actin and expressed as optical density (O.D.).

Image acquisition and analyses

Image acquisition was performed using a Nikon C80i microscope equipped with a motorized stage (Ludl) attached to Microfire CCD color camera (Optronics). The density and coverage area of FJB^+, GFP^+, IBA-1^+ cells were quantified by unbiased stereological analysis [6] using Stereo Investigator software (version 6.02.1, MicroBrightfield) reported to total brain area. For each animal, 4 sections (+ 1,42 mm; + 0,26 mm; -1.22 mm; -2.18 mm from the bregma according to a stereotaxic atlas, Paxinos and Franklin, 2nd edition) were analyzed. For the IgG staining, images of sections were taken with a molecular imager (Storm 860, Molecular Dynamic). IgG extravasation was expressed as the percentage

of IgG coverage area reported to total brain area.

SUPPLEMENTAL REFERENCES

1. Lampron A, Lessard M, Rivest S. Effects of Myeloablation, Peripheral Chimerism and Whole Body Irradiation on the Entry of Bone Marrow-Derived Cells Into the Brain. *Cell Transplant*. 2011;21:1149–1159

2. Karatas H, Erdener SE, Gursoy-Ozdemir Y, Gurer G, Soylemezoglu F, Dunn AK, Dalkara T. Thrombotic distal middle cerebral artery occlusion produced by topical FeCl(3) application: a novel model suitable for intravital microscopy and thrombolysis studies. *J. Cereb. Blood Flow Metab*. 2011;31:1452–1460.

3. Lampron A, Pimentel-Coelho PM, Rivest S. Migration of bone marrow-derived cells into the central nervous system in models of neurodegeneration. *J. Comp. Neurol.* 2013;521:3863–3876.

4. ElAli A, Bordeleau M, Thériault P, Filali M, Lampron A, Rivest S. Tissue-Plasminogen Activator Attenuates Alzheimer's Disease-Related Pathology Development in APPswe/PS1 Mice. *Neuropsychopharmacology*. 2015;41:1297–1307.

5. Turrin NP, Rivest S. Molecular and Cellular Immune Mediators of Neuroprotection. *Mol. Neurobiol.* 2006;34:221–242.

6. Boissonneault V, Filali M, Lessard M, Relton J, Wong G, Rivest S. Powerful beneficial effects of macrophage colony-stimulating factor on beta-amyloid deposition and cognitive impairment in Alzheimer's disease. *Brain*. 2009;132:1078–1092.
